# Efficacy evaluation of novel organic iron complexes in laying hens: effects on laying performance, egg quality, egg iron content, and blood biochemical parameters

**DOI:** 10.5713/ab.22.0086

**Published:** 2022-09-07

**Authors:** Jiuai Cao, Jiaming Zhu, Qin Zhou, Luyuan Zhao, Chenhao Zou, Yanshan Guo, Brian Curtin, Fei Ji, Bing Liu, Dongyou Yu

**Affiliations:** 1 College of Animal Sciences, Zhejiang University, Hangzhou, Zhejiang 310058, China; 2 Hainan Institute, Zhejiang University, Sanya 572025, China; 3 Zinpro Corporation, Eden Prairie, MN 55344, USA

**Keywords:** Blood Biochemical Parameters, Egg Yolk Iron, Iron Amino Acid Complex, Laying Hen, Performance

## Abstract

**Objective:**

This study was conducted to determine the optimal dose of novel iron amino acid complexes (Fe-Lys-Glu) by measuring laying performance, egg quality, egg iron (Fe) concentrations, and blood biochemical parameters in laying hens.

**Methods:**

A total of 1,260 18-week-old healthy Beijing White laying hens were randomly divided into 7 groups with 12 replicates of 15 birds each. After a 2-wk acclimation to the basal diet, hens were fed diets supplemented with 0 (negative control, the analyzed innate iron content was 75.06 mg/kg), 15, 30, 45, 60, and 75 mg Fe/kg as Fe-Lys-Glu or 45 mg Fe/kg from FeSO_4_ (positive control) for 24 wk.

**Results:**

Results showed that compared with the negative and positive control groups, dietary supplementation with 30 to 75 mg Fe/kg from Fe-Lys-Glu significantly (linear and quadratic, p<0.05) increased the laying rate (LR) and average daily egg weight (ADEW); hens administered 45 to 75 mg Fe/kg as Fe-Lys-Glu showed a remarkable (linear, p<0.05) decrease in feed conversion ratio. There were no significant differences among all groups in egg quality. The iron concentrations in egg yolk and serum were elevated by increasing Fe-Lys-Glu levels, and the highest iron content was found in 75 mg Fe/kg group. In addition, hens fed 45 mg Fe/kg from Fe-Lys-Glu had (linear and quadratic, p<0.05) higher yolk Fe contents than that with the same dosage of FeSO_4_ supplementation. The red blood cell (RBC) count and hemoglobin content (linear and quadratic, p<0.05) increased obviously in the groups fed with 30 to 75 mg Fe/kg as Fe-Lys-Glu in comparison with the control group. Fe-Lys-Glu supplementation also (linear and quadratic, p<0.05) enhanced the activity of copper/zinc-superoxide dismutase (Cu/Zn-SOD) in serum, as a result, the serum malonaldehyde content (linear and quadratic, p<0.05) decreased in hens received 60 to 75 mg Fe/kg as Fe-Lys-Glu.

**Conclusion:**

Supplementation Fe-Lys-Glu in laying hens could substitute for FeSO_4_ and the optimal additive levels of Fe-Lys-Glu are 45 mg Fe/kg in layers diets based on the quadratic regression analysis of LR, ADEW, RBC, and Cu/Zn-SOD.

## INTRODUCTION

Iron (Fe) is an indispensable element for organisms that plays crucial roles in several fundamental metabolic processes, including erythropoiesis, oxygen transport, deoxyribonucleic acid (DNA) synthesis, energy metabolism, mitochondrial electron transport, immune-protection, and cognitive function [1–4]. Iron should be maintained at an appropriate level which is required to satisfy the metabolic needs and specialized functions for animals [5], while iron deficiency causes anemia, abnormal development, and severe deficiency could lead to death [6,7]. The strategy to reduce iron deficiency is iron supplementation in feed [8]. Traditionally, breeding hens are fed with a form of inorganic iron salts in the diets, such as ferrous sulfates (FeSO_4_) [9]. However, the inorganic iron sources have several defects, like low bioavailability, high oxidation, and excretion, which can pollute the environment [10]. As a result, chelates or complexes of iron with amino acid or protein, organic iron compounds are being developed as substitute for inorganic iron. Meanwhile, it was reported that organic trace elements have higher bioavailability compared with inorganic microelements [11]. The relative bioavailability of organic mineral elements was determined by the chelation strength and the greater chelation strength indicates higher biological value and beneficial effects to laying hens [12,13]. The studies in literature mainly focused on ferric glycine (Fe-Gly) and ferric methionine (Fe-Met). Previous study found that the absorption rate of iron in Fe-Gly was twice of that in FeSO_4_ [14]. Compared with the same dosage of FeSO_4_, dietary supplementation with Fe-Gly (60 mg/kg) effectively improved egg weight and increased the enrichment of iron in egg albumen and yolk [15]. Another study also reported that Fe-Gly was superior to FeSO_4_ in the content of iron in serum and eggshell, albumen, and yolk [6]. In addition, these results indicate that low-dose (about 60 mg/kg) of Fe-Gly could replace inorganic iron, with preferable impacts on egg quality and iron deposition. Fe-Met has been confirmed to be a safe and effective source of iron, with a higher biological value than FeSO_4_ for piglets [11]. However, a new organic form of iron active compounds, iron amino acid complexes (1:1 complex of Fe lysine and Fe glutamic acid, Fe-Lys-Glu), in laying hens has not been studied.

Therefore, the purpose of this study was to investigate the effects of dietary Fe-Lys-Glu level on laying performance, egg quality, egg iron content, and blood biochemical parameters. The quadratic equation regression analysis was used to determine the optimal supplemental of Fe-Lys-Glu. Furthermore, we examined whether Fe-Lys-Glu could substitute FeSO_4_ in laying hens.

## MATERIALS AND METHODS

### Animal care

The use of experimental animals and all procedures were performed in accordance with the Chinese Guidelines for Animal Welfare and approved by the Animal Care and Use Committee of Zhejiang University.

### Experimental design and management

A total of 1,260 18-week-old Beijing White laying hens with similar body weights were randomly allocated into 7 groups with 12 replicates (15 birds in each replicate). After adapting to the basal diet (Table 1) and environment for 2 wk, the hens were fed with 0 (negative control, NC), 15, 30, 45, 60, and 75 mg Fe/kg of diet in Fe-Lys-Glu form (15% Zinpro-Fe 150, provided by Zinpro Corporation, Eden Prairie, MN, USA) or 45 mg Fe/kg as FeSO_4_ (positive control, provided by Zhejiang Weimeng Feed Technology Co., Ltd, Hangzhou, China) for 24 wk. The basal diet was formulated depending on the protein, energy, and mineral requirements (NRC, 1994) and the Chinese Feeding Standard of Chicken (NY/T 33-2004) without adding exogenous iron (analyzed iron content 75.06 mg/kg). Every three birds were placed in a cage (0.50 m length×0.45 m width×0.40 m height) with a 16 L:8 D photoperiod with 15 to 20 lx light intensity during the entire experimental period. Feed and water were provided *ad libitum*. The housing temperature and relative humidity were maintained at 24°C±3°C and 50% to 60%, respectively. Immunization and disinfection were carried out according to routine methods.

### Sample collection and analytical determination

#### Chemical component analysis of feed sample

The feed samples were obtained by quartering method and mixed evenly. Then, 500 g feed of each group was put into the labeled sample bag and stored in a refrigerator at −20°C for the determination of amino acids, crude protein, calcium, phosphorus, and iron. The contents of amino acids and crude protein in diets were determined according to the methods of the Association of Official Analytical Chemists (AOAC) [16] and the iron and calcium contents in feed samples were analyzed using Atomic Absorption Spectroscopy (AAS) according to AOAC [16]. The phosphorus content of the basal diets was determined with the method of previous study [17].

#### Laying performance

Egg numbers and total egg weight of each replicate were recorded daily, and the feed consumption was weighed once a week. Based on the collected data, the laying rate (LR), average daily egg mass, average daily feed intake (ADFI), and feed conversion ratio (FCR) were calculated.

#### Determination of egg quality

At the end of the trial period, 6 eggs per replicate were randomly collected to determine the egg quality, including albumen height, Haugh unit, yolk color, eggshell strength, eggshell thickness, egg yolk, albumen, and eggshell ratio. The determination methods were reported previously [18].

Iron content in egg yolk at the end of 12 and 24 wk, 6 eggs per replicate were randomly selected to measure the Fe concentrations in egg yolk using AAS according to the method described by Revy et al [19]. The results were expressed as mg/kg fresh weight.

#### Blood biochemical parameters

At the end of the trial, one bird was chosen in random from each replicate (n = 12). After fasting for 12 h, blood samples from the wing vein were collected. One part of the blood was used to determine blood indexes including red blood cells (RBC), hemoglobin (HGB), and hematocrit (HCT) by a Sysmex microcell counter CL-180 automated hematology analyzer (Sysmex Company, Lincolnshire, IL, USA). And the other part of the blood was then centrifugated at 3,500 r/min for 10 min at 4°C to obtain serum samples. The serum samples were used to detect the antioxidant capacity including copper/zinc-superoxide dismutase (Cu/Zn-SOD), manganese-SOD (Mn-SOD), and malondialdehyde (MDA) by kits (Nanjing Jiancheng Bioengineering Institute, Nanjing, China). Another 1 mL serum was mixed with 9 times volume of 10% trichloroacetic acid, and then centrifugated at 3,500 r/min for 10 min, the supernatant collected and brought up to constant volume of 10 mL by the addition of 1.2 mol/L nitric acid. The iron content of the supernatant was measured by AAS and was consistent with the determination of yolk iron [19].

### Statistical analysis

All data were analyzed with general linear model procedure of SPSS software (SPSS 21.0, SPSS Inc., Chicago, IL, USA) and multiple comparisons between the groups were performed by least significant difference test. Besides, the first six groups of hens fed with 0, 15, 30, 45, 60, and 75 mg Fe/kg Fe-Lys-Glu in diet of data were analyzed by linear and quadratic regression analysis. p<0.05 was considered statistically significant.

## RESULTS

### Laying performance

Compared with the negative control group, 15 to 75 mg Fe/kg diet from Fe-Lys-Glu supplementation significantly (linear and quadratic, p<0.05) increased LR (Table 2), and the hens fed 30 to 75 mg /kg diet-supplemented diet had higher ADEW. Hens that received 45 to 75 mg Fe/kg as Fe-Lys-Glu (linear, p<0.05) decreased FCR remarkably compared with the hens in the negative control group. Compared with the positive control, hens fed with 45 mg Fe/kg from Fe-Lys-Glu had obviously increased LR and decreased FCR (p<0.05). Moreover, even though the added iron level (30 mg/kg) was below that of inorganic iron group (45 mg Fe/kg), it still showed better laying performance. According to the equation of regression (Table 7), the Fe supplementation value was 48.57 to 49.5 mg/kg when the LR and ADEW reached the maximum response.

### Egg quality

There were no significant differences in any parameters (Table 3), including albumen height, Haugh unit, yolk color, eggshell strength, eggshell thickness, yolk, albumen, and eggshell ratio among treatments.

### Iron content in egg yolk and serum

Data presented in Table 4 indicates that hens fed with 15 to 75 mg Fe/kg as Fe-Lys-Glu (linear and quadratic, p<0.05) had elevated contents of iron in egg yolk and serum at 12 wk and 24 wk in comparison with the negative control group. The iron concentrations in egg yolk and serum are proportional to the additive dosage of Fe-Lys-Glu. Hens in 45 mg/kg Fe from Fe-Lys-Glu-supplemented group had higher (p<0.05) egg yolk and serum iron contents than in 45 mg/kg from FeSO_4_-added group. Interestingly, the lowest dose of Fe-Lys-Glu (15 mg Fe/kg) had more enrichment of iron in egg yolk than FeSO_4_ group with 45 mg Fe/kg.

### Blood biochemical parameters

The effects of dietary Fe-Lys-Glu supplementation on biochemical parameters of laying hens are presented in Table 5. Compared with the negative control group, the groups of 30 to 75 mg Fe/kg as Fe-Lys-Glu diet had (linear and quadratic, p<0.05) elevated RBC count and HGB content. The supplemental groups of 60 to 75 mg Fe/kg from Fe-Lys-Glu generated the highest HGB content and RBC count. For these blood parameters, the same dose of Fe-Lys-Glu (45 mg Fe/kg) showed better effects than that of FeSO_4_. There was no statistically significant difference found in hematological tests among all the treatments (HCT). Through the quadratic regression analysis of RBC (Table 7), we could find that the Fe supplementation level was 67.82 mg/kg when the indictor reached the maximum response.

### Antioxidant capability

As shown in Table 6, Fe-Lys-Glu diets obviously (linear and quadratic, p<0.05) enhanced the activity of serum Cu/Zn-SOD in comparison with the negative control group. Birds fed with 60 to 75 mg Fe/kg from Fe-Lys-Glu presented a remarkable (linear and quadratic, p<0.05) reduction in the content of MDA in serum. In general, the higher the Fe-Lys-Glu addition level, the stronger the antioxidant capacity (higher Cu/Zn-SOD activity and lower MDA content) of hens. Fe-Lys-Glu supplementation did not affect the activity of Mn-SOD in serum of layers. The Fe supplementation value was 70.75 mg/kg when the Cu/Zn-SOD reached the maximum response (Table 7).

## DISCUSSION

### Laying performance

Poultry are sensitive to iron content in their diets and iron deficiency greatly affects their growth [20]. Therefore, it is necessary to explore the optimal level of Fe-Lys-Glu, a new organic iron source in laying hens. Some early studies found that the bioavailability of Fe-Gly was at least 2 times more than FeSO_4_ [14,21,22]. Supplementing Fe-Gly in laying hens increased LR and egg weight compared with FeSO_4_, while ADFI and FCR showed no differences [23]. Adding Fe-Gly in the basal diet enhanced performance of hens and decreased the abnormal egg rate in contrast to inorganic ferrous [6]. Compared with FeSO_4_, sows supplemented with Fe-Gly did not have a negative effect on the performance of piglets, while reducing iron excretion in feces [24,25]. The above results show that Fe-Gly had a greater absorption rate than FeSO_4_ in animal production. The present study found a remarkable increase in LR and ADEW of layers fed with 15 to 75 mg Fe/kg and 30 to 75 mg Fe/kg as Fe-Lys-Glu compared with diet without adding exogenous iron. Additionally, FCR of hens in the groups of 45, 60, 75 mg Fe/kg from Fe-Lys-Glu was decreased markedly in comparison with the group without adding exogenous iron. It is because the organic source of iron elevates the absorption of mineral elements in hens by reducing the ability to bind with nutrients or antinutritional factors, thereby improving the laying performance [26]. However, when the dosage of Fe-Lys-Glu reached 75 mg Fe/kg, the laying performance showed a downward trend compared with 60 mg Fe/kg. This suggests that optimal dosage of Fe-Lys-Glu should not exceeding 60 mg Fe/kg. Additionally, even though the addition level of Fe-Lys-Glu was lower than that of FeSO_4_, it still showed better laying performance. It indicates that Fe-Lys-Glu has greater bioavailability than inorganic ferrous. Our results suggest that the organic iron source (Fe-Lys-Glu) could potentially be used as a substitute for FeSO_4_ and applied to the practical production of laying hens. Through the quadratic regression analysis of LR and ADEW, we can see that when the supplemental dose of Fe-Lys-Glu reached 48.57 and 49.5 mg Fe/kg, the laying performance of laying hens reached the best.

### Egg quality

Fe-Gly supplementation presented a positive effect on eggshell thickness and Haugh unit [27]. Eggshell color was prominently affected by supplementing Fe-Met and iron-soy proteinate (Fe-SP) [28]. Other study indicated that supplementation of Fe-Gly distinctly increased the Haugh unit and albumen height, compared with the control with supplementing no ferric glycine, while there were no visible differences among the groups of FeSO_4_ and Fe-Gly with the same supplementation level [15]. Although our study showed that adding Fe-Lys-Glu in the diets had no significant effect any parameters of egg quality at wk 24 of the experiment, Fe-Lys-Glu showed a tendency to improve egg quality.

### Iron concentration of egg yolk

Phosvitin is the main ferritic carrier protein in yolk [29] and accounts for a proportion of about 60% of the total phosphoproteins in yolk which are mainly made up of phosphoserine [30]. Phosphoserine binds to iron and calcium strongly and inhibits the formation of insoluble iron complexes or calcium phosphates, resulting in improving the bioavailability of iron and calcium and restraining lipid oxidation [30]. It means that providing a high bioavailability diet could increase the iron absorption rates in organisms and the storage of iron in egg yolk. Several studies reported that iron contents in egg yolk were affected by different sources of iron and experiment period [6,9]. They found that adding Fe-amino acids (Fe-AA) to diets distinctly increased the enrichment of iron in egg yolk, compared with adding FeSO_4_. The content of iron obviously increased as the experiments went on. We found that additive level of Fe-Lys-Glu and experimental period effectively affected the deposition of iron in egg yolk, which was consistent with previous studies. It also confirms the above conclusion of laying performance: Fe-Lys-Glu has a higher biological efficacy than FeSO_4_.

### Iron biochemical parameters

It is well known that hematological and serum biochemical parameters of animals are important indictors for basic metabolism, performance, and health [31]. Iron is necessary for several metabolic processes in organisms and cells. Sufficient plasma iron concentration is the key to iron supply and balance [32]. The biological response to iron in organisms is usually evaluated by several parameters including RBC, HGB, and HCT [33]. Iron is a necessary element for production of RBC and synthesis of HGB [4]. The HGB content and HCT are commonly used to estimate iron status and affected by different sources of iron, while low level of them indicates a nutritional anemia [10,34]. It was reported that adding Fe-Gly in diet increased serum iron, RBC count, HGB content, and HCT of piglets compared with negative control and FeSO_4_ groups [35]. Similar results were found in pregnant sows [36]. The results may demonstrate that organic iron has higher biological value than inorganic iron. Our study presented an upward tendency in iron content of serum with increasing dosage of Fe-Lys-Glu. The process of experiment also affected serum iron levels. The RBC count and HGB content were significantly increased in chickens fed with 30 to 75 mg Fe/kg from Fe-Lys-Glu diet.

### Antioxidant capacity

The bioactivity of iron depends on the ability to transfer electrons, allowing it to be involved in many reactions like the Fenton reaction [2]. However, iron can damage tissues arising from catalyzing the generation of free radicals and reactive oxygen species through Fenton reaction under aerobic conditions [5]. The resulting free radicals could attack cytomembrane, proteins and DNA [37]. The Cu/Zn-SOD is an antioxidant enzyme which primarily regulates oxidate stress produced by mitochondrial and cytosolic superoxide, while Mn-SOD merely exists in mitochondria [38]. The SOD could spontaneously decompose superoxide radicals into hydrogen peroxide (H_2_O_2_) by dismutation [39]. Superfluous free radicals act on lipids inducing a process of lipid peroxidation which produces MDA ultimately [40]. Several studies have shown that supplementing organic source of iron could improve antioxidant capacity of the body. The activities of T-SOD and Cu/Zn-SOD were raised along with the growth of additive dosage of Fe-Gly [23]. The Fe-Gly supplementation effectively increased the activities of SOD and decreased the content of MDA in serum of layers at 9 wk and 12 wk of the experiment [6]. Cu/Zn-SOD activity in liver of broilers was increased by the addition of Fe-Gly to diets [41]. Above results suggest that Fe-Gly can be used as an organic iron source replacing FeSO_4_. In this study, we found that adding 45 to 60 mg Fe/kg of Fe-Lys-Glu in the diets as organic iron source significantly improved antioxidant capacity of laying hens by enhancing Cu/Zn-SOD activities and decreasing MDA content in serum, which is consistent with these studies. Dietary supplementation of 75 mg Fe/kg as Fe-Lys-Glu had the same effect on antioxidant capacity of hens as that of 60 mg Fe/kg, so the optimal level of Fe-Lys-Glu should not be greater than 60 mg Fe/kg according to laying performance and antioxidant capacity of hens. Thus, our results imply that Fe-Lys-Glu can be considered as a good source of organic iron for replacing FeSO_4_.

## CONCLUSION

The present study demonstrated that Fe-Lys-Glu was an effective iron source that could substitute for FeSO_4_ in laying hen diets. Dietary Fe-Lys-Glu supplementation is beneficial to improve the laying performance, antioxidant ability, and yolk Fe content of laying hens in varying degrees. Based on the quadratic model analyses, supplemental 45 mg Fe/kg via Fe-Lys-Glu could provide the optimum laying performance (LR and ADEW). It is recommended that the dosage of Fe-Lys-Glu should not be higher than 60 mg Fe/kg.

## Figures and Tables

**Table 1:** Ingredient composition and nutrient levels of the basal diet for the hens (air-dry basis)

Items	Content (%)
Ingredients	
Corn	64.15
Soybean meal	25.00
Calcium carbonate	8.00
Calcium hydrogen phosphate	1.40
Sodium chloride	0.30
DL-Methionine	0.15
Premix^1)^	1.00
Total	100.00
Nutrient levels^2)^	
Metabolizable energy (kcal/kg)	2,680
Crude protein	16.59
Lysine	0.84
Methionine	0.44
Cysteine+Methionine	0.70
Calcium	3.36
Total phosphorus	0.66
Non-phytate phosphorus	0.54
Fe (mg/kg)	75.06

1) Premix provided per kilogram of diet: vitamin A, 12,500 IU; vitamin D_3_, 4,125 IU; vitamin E, 30 IU; vitamin K_3_, 2 mg; thiamine, 1 mg; riboflavin, 8.5 mg; calcium pantothenate, 50 mg; nicotinic acid, 32.5 mg; vitamin B_6_, 8 mg; folic acid, 5 mg; vitamin B_12_, 5 mg; biotin, 1 mg; choline chloride, 600 mg; Iodine, 0.35 mg; Copper, 10 mg; Manganese, 80 mg; Zinc, 80 mg; Selenium, 0.3 mg.

2) Except for metabolizable energy and non-phytate phosphorus, all the rest were measured values.

**Table 2:** Effects of dietary Fe-Lys-Glu supplementation on performance of laying hens

Items	Supplementary level of Fe-Lys-Glu (mg Fe/kg)						FeSO_4_	SEM	p-value		
		
	0	15	30	45	60	75	45		Treatment	Linear	Quadratic
LR (%)	88.35^d^	91.14^bc^	93.18^ab^	93.39^ab^	94.05^a^	93.21^ab^	90.58^cd^	0.840	<0.001	<0.001	0.006
ADEW (g/hen/d)	47.24^c^	48.21^bc^	49.11^ab^	49.70^ab^	50.06^a^	49.13^ab^	48.15^bc^	0.639	0.038	0.004	0.044
ADFI (g/hen/d)	106.75	108.24	108.81	105.75	107.07	106.47	106.75	0.861	0.190	0.275	0.347
FCR	2.27^a^	2.25^a^	2.22^ab^	2.14^c^	2.13^c^	2.16^bc^	2.21^ab^	0.025	<0.001	<0.001	0.105

LR, egg production rate; ADEW, average daily egg weight; ADFI, average daily feed intake; FCR, feed convention ratio.

a–d The means in the same row with the same superscripts or without letters do not differ significantly (p>0.05), while the distinct superscripts show significant differences (p<0.05).

**Table 3:** Effects of dietary Fe-Lys-Glu supplementation on egg quality of laying hens

Items	Supplementary level of Fe-Lys-Glu (mg Fe/kg)						FeSO_4_	SEM	p-value		
		
	0	15	30	45	60	75	45		Treatment	Linear	Quadratic
Albumen height (mm)	6.84	6.74	6.83	6.75	6.84	6.77	6.53	0.202	0.938	0.930	0.928
Haugh unit	83.74	86.62	85.08	84.36	84.94	83.79	80.55	1.672	0.294	0.693	0.445
Yolk color	6.33	6.17	6.42	6.25	6.50	6.67	6.25	0.274	0.879	0.286	0.515
Eggshell strength (kg/m^2^)	3.38	3.48	3.46	3.62	3.60	3.60	3.36	0.112	0.503	0.114	0.654
Eggshell thickness (mm)	0.33	0.33	0.35	0.34	0.33	0.34	0.33	0.008	0.687	0.686	0.428
Yolk ratio (%)	25.27	22.19	22.95	23.80	25.82	25.86	25.65	1.533	0.450	0.268	0.157
Albumen ratio (%)	60.56	60.64	62.64	62.25	61.63	62.42	61.04	0.783	0.305	0.068	0.330
Eggshell ratio (%)	14.17	17.17	14.42	13.95	12.55	11.72	13.31	1.275	0.102	0.019	0.260

SEM, standard error of the mean.

**Table 4:** Effects of dietary Fe-Lys-Glu supplementation on the concentrations of iron in egg yolk and serum

Items	Supplementary level of Fe-Lys-Glu (mg Fe/kg)						FeSO_4_	SEM	p-value		
		
	0	15	30	45	60	75	45		Treatment	Linear	Quadratic
Iron content in egg yolk (mg/kg fresh weight)											
12 wk	51.98^e^	54.17^cd^	54.83^c^	57.37^b^	57.43^b^	63.41^a^	53.50^d^	0.396	<0.001	<0.001	<0.001
24 wk	56.39^d^	61.50^bc^	61.93^b^	63.42^a^	63.31^a^	63.19^a^	61.02^c^	0.317	<0.001	<0.001	0.080
Iron content in serum (mg/L)											
12 wk	1.92^d^	2.13^c^	2.11^c^	2.32^b^	2.48^a^	2.55^a^	2.18^c^	0.027	<0.001	<0.001	0.981
24 wk	2.21^e^	2.41^d^	2.60^c^	2.78^b^	2.97^a^	3.11^a^	2.47^cd^	0.052	<0.001	<0.001	0.600

SEM, standard error of the mean.

a–e The means in the same row with the same superscripts or without letters do not differ significantly (p>0.05), while the distinct superscripts show significant differences (p<0.05).

**Table 5:** Effects of dietary Fe-Lys-Glu supplementation on blood biochemical parameters of laying hens

Items	Supplementary level of Fe-Lys-Glu (mg Fe/kg)						FeSO_4_	SEM	p-value		
		
	0	15	30	45	60	75	45		Treatment	Linear	Quadratic
RBC (10^12^/L)	2.73^d^	2.88^cd^	3.00^abc^	3.16^ab^	3.09^ab^	3.17^a^	2.70^d^	0.066	<0.001	<0.001	0.004
HGB (g/L)	208.81^c^	214.48^bc^	221.51^b^	233.57^a^	241.64^a^	238.87^a^	207.18^c^	3.400	<0.001	<0.001	0.179
HCT (%)	38.44	37.89	38.28	38.14	37.06	37.29	38.87	0.683	0.517	0.092	0.710

SEM, standard error of the mean; RBC, red blood cells; HGB, hemoglobin; HCT, hematocrit.

a–d The means in the same row with the same superscripts or without letters do not differ significantly (p>0.05), while the distinct superscripts show significant differences (p<0.05).

**Table 6:** Effects of dietary Fe-Lys-Glu supplementation on serum antioxidant capability of laying hens

Items	Supplementary level of Fe-Lys-Glu (mg Fe/kg)						FeSO_4_	SEM	p-value		
		
	0	15	30	45	60	75	45		Treatment	Linear	Quadratic
Mn-SOD (U/mL)	164.36	173.81	179.83	177.49	175.97	161.69	152.67	9.361	0.345	0.912	0.105
Cu/Zn-SOD (U/mL)	75.21^d^	80.85^c^	82.06^bc^	83.40^ab^	84.06^a^	84.23^a^	82.94^ab^	0.652	<0.001	<0.001	<0.001
MDA (nmol/mL)	5.32^a^	5.29^a^	5.30^a^	5.27^ab^	5.21^b^	5.14^c^	5.28^a^	0.021	<0.001	<0.001	0.015

SEM, standard error of the mean; Mn-SOD, manganese-superoxide dismutase; Cu/Zn-SOD, copper/Zinc-superoxide dismutase; MDA, malondialdehyde.

a–d The means in the same row with the same superscripts or without letters do not differ significantly (p>0.05), while the distinct superscripts show significant differences (p<0.05).

**Table 7:** Estimated change of the sensitive indices in laying hens with Fe-Lys-Glu supplementation

Variation	Equation of regression	Estimated Fe supplementation	Estimated maximum response	p-values	R^2^
LR	y = 88.436+0.204x–0.0021x^2^	48.57	93.39	0.006	0.294
ADEW	y = 47.103+0.099x–0.001x^2^	49.5	49.55	0.044	0.167
RBC	y = 2.722+0.012x–8.847e^−05^x^2^	67.82	3.13	0.004	0.556
Iron content in yolk (12 wk)	y = 52.551+0.034–0.001x^2^	17.00	52.84	<0.001	0.817
Cu/Zn-SOD	y = 75.870+0.283x–0.002x^2^	70.75	85.88	<0.001	0.641
MDA	y = 5.311+0.001x–3.790e^−05^x^2^	13.19	5.32	0.015	0.435

LR, laying rate; ADEW, average daily egg weight; RBC, red blood cell; Cu/Zn-SOD, Copper/Zinc-superoxide dismutase; MDA, malondialdehyde.

The equation of regression is y = a+bx+cx^2^. Y means the laying performance, RBC, iron content in egg yolk (12 wk) and antioxidant indices, and the Fe supplementation was estimated when the concentration of the above indicators reached the maximum response.
